# Development of ^111^In-labeled porphyrins for SPECT imaging

**Published:** 2014

**Authors:** Shaghayegh Sadeghi, Mohammad Mirzaei, Mohammad Rahimi, Amir R. Jalilian

**Affiliations:** Radiation Application Research School, Nuclear Science and Technology Research Institute (NSTRI), Iran

**Keywords:** Hydroxy phenyl porphyrins, ^111^In, Biodistribution, Radiolabeling, SPECT

## Abstract

**Objective(s):**

The aim of this research was the development of ^111^In-labeled porphyrins as possible radiopharmaceuticals for the imaging of tumors.

**Methods:**

Ligands, 5, 10, 15, 20-tetrakis (3, 5-dihydroxyphenyl) porphyrin) (TDHPP), 5, 10, 15, 20-tetrakis (4-hydroxyphenyl) porphyrin (THPP) and 5, 10, 15, 20-tetrakis (3,4-dimethoxyphenyl) porphyrin) (TDMPP) were labeled with ^111^InCl3 (produced from proton bombardment of natCd target) in 60 min at 80 ºC. Quality control of labeled compounds was performed via RTLC and HPLC followed by stability studies in final formulation and presence of human serum at 37 ºC for 48 h as well as partition coefficient determination. The biodistribution studies performed using tissue dissection and SPECT imaging up to 24h.

**Results:**

The complexes were prepared with more than 99% radiochemical purity (HPLC and RTLC) and high stability to 48 h. Partition coefficients (calculated as log P) for ^111^In-TDHPP, ^111^In-THPP and ^111^In-TDMPP were 0.88, 0.8 and 1.63 respectively.

**Conclusion:**

Due to urinary excretion with fast clearance for ^111^In-TDMPP, this complex is probably a suitable candidate for considering as a possible tumor imaging agent.

## Introduction

Porphyrins can be appropriate ligands for designing metal complex including radio-pharmaceuticals because porphyrins and their related compounds are used as tumor seeking drugs, especially as photosensitizers in the photodynamic therapy (PDT) of cancer (1, 2) where the combination of light and photosensitizer, generates active oxygen species near the tumor, to damage the malfunctioned tissues. Varieties of porphyrins including anionic porphyrins (3), hematoporphyrins (4), cationic porphyrins (5) and phethalocyanines (6) have been successfully used for tumor treatment.

Porphyrins taken into the blood circulation as tumor localizing agents are transported to target tissue by human serum albumin (HSA) and other plasma proteins such as low and high-density lipoproteins (7). Other investigators have suggested that porphyrin accumulation in the tumor is related to low-density lipoprotein (LDL) uptake that occurs through receptor-mediated endocytosis (8).

Meso tetrakis (4-hydroxyphenyl) porphyrin (THPP) has been used as a potential molecule in PDT with high photosensitivity for cancer treatment, leading to the destruction of intrahepatic tumors with better efficacy and fewer side effects (9).

Several investigators have reported the synthesis and radiolabeling of wide varieties of porphyrin derivatives with various types of peripheral moieties with several important medical radionuclides for developing an ideal tumor localizing agent (10-15). However, none of these radiolabeled porphyrins have succeeded as a popular regular product. In-111 is a cyclotron produced radionuclide, decaying by electron capture (EC) with subsequent emission of gamma photons of 173 and 247 keV (89% and 94% intensity, respectively), is widely used in gamma scintigraphy. It is reported that ^111^In-labeled porphyrins exhibit specific accumulation in tumor tissues (16, 17).

**Figure 1:**
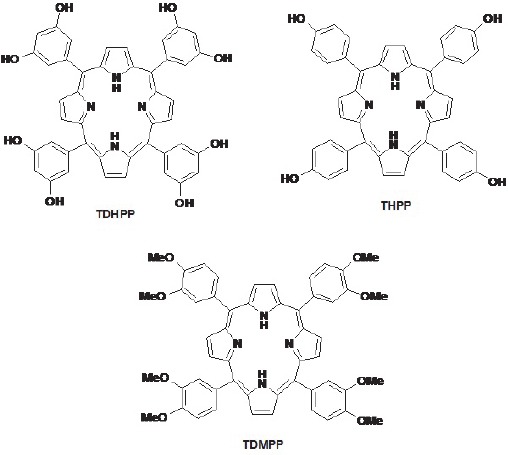
Chemical structures for ligands used in this work

Our attempt was to prepare a series of water soluble suitable radiolabeled porphyrins for tumor diagnosis, due to the availability of In-111. Structures of ligands used in this study are shown in Figure 1.

In this research, synthesis, radiolabeling, partition coefficient, quality control and bio-distribution studies using SPECT and scarification of ^111^In-TDHPP, ^111^In-THPP and ^111^In-TDMPP in wild-type rats are reported.

## Methods

In-111 was produced at the Agricultural, Medical and Industrial Research School (AMIRS), 30 MeV cyclotron (Cyclone-30, IBA) using ^nat^Cd(p,x)^111^In reaction. Natural cadmium sulfate with a purity of more than 99% was obtained from Merck Co. Germany. All chemicals were purchased from Sigma-Aldrich Chemical Co. U.K. Radio-chromatography was performed by Whatman paper using a thin layer chromatography scanner, Bioscan AR2000, Paris, France. Analytical HPLC to determine the specific activity was performed by a Shimadzu LC-10AT, armed with two detector systems, flow scintillation analyzer (Packard-150 TR) and UV-visible (Shimadzu) using Whatman Partisphere C-18 column 250 × 4.6 mm (Whatman Co. NJ, USA). Calculations were based on the 172 keV peak for In-111. All values were expressed as mean ± standard deviation (Mean ± SD) and the data were compared using student T-test. Animal studies were performed in accordance with the United Kingdom Biological Council's Guidelines on the Use of Living Animals in Scientific Investigations, second edition.

### Electroplating of the natural Cd targets:

In order to prepare Cd targets for the production, cadmium electroplating was performed over a copper surface was performed according to the previously reported method (18). Cadmium was electroplated over the copper backing according to the method given in the literature (19). A mixture of CdSO_4_.8/3H_2_O, KCN, BrijTM detergent solution and traces of hydrazine hydrate with a final volume of 450 ml double-distilled water (DDH_2_O) at pH=13 was used as the electroplating bath (constant current: 320 mA, stirring rate 780 rpm, time 0.5 h). After the deposition of an about 500 mg cadmium layer, the targets were wrapped in Parafilm^®^ coatings to avoid atmospheric oxygen exposure. Finally, the target was sent for irradiation.

### Production and quality control of ^111^InCl_3_ solution

Indium-111 chloride was prepared by 22 MeV proton bombardment of the cadmium target at a 30 MeV cyclotron, with a current of 100 μA for 48 min (80 μAh). After dissolution of the irradiated target by conc. HBr, the solution was passed through a cation exchange dowex 50×8 resin, pre-conditioned by 25 ml of conc. HBr. The resin was then washed by HBr conc. solution (50 ml). In order to remove the undesired impurities of Cd and Cu, the resin was totally washed with DDH_2_O. Indium-111 was eluted with 1 N HCl (25 ml) as ^111^InCl_3_ for labeling use.

### Quality control of the product

**Control of Radionuclide purity:** Gamma spectroscopy of the final sample was carried out counting in an HPGe detector coupled to a Canberra multi-channel analyzer for 1000 seconds.

**Chemical purity control:** This step was carried out to ensure that the amounts of cadmium and copper ions resulting from the target material and backing in the final product are acceptable regarding internationally accepted limits. Chemical purity was checked by differential-pulsed anodic stripping polaro-graphy. The detection limit of our system was 0.1 ppm for both cadmium and copper ions.

### Preparation and quality control of radiolabeled porphyrins

Complexion of In-111 with porphyrins was carried out by using acidic solution of ^111^InCl_3_, acetate buffer and porphyrins in absolute ethanol. The reaction was performed by adding acidic solution (100 µl) of ^111^InCl_3_ (185 MBq, 5 mCi) to a 5 mL-borosilicate vial. The solution was heated under a flow of nitrogen till it was dried at 50^º^C. A volume (100 µl) of porphyrin dissolved in absolute ethanol (20 mg/ml, 290-300 nmol) was transferred to the vial. The pH was adjusted to 5.5-7 by adding 2000 µl of acetate buffer. Resulting solution was stirred at 25 °C for 30–60 min at pH=5. Radiochemical purity of the solution was measured by RTLC and HPLC. Radio thin layer chromatography was done with 10% ammonium acetate: methanol (1:1) mixture as mobile phase and chromatography Whatman No. 2 paper as stationary phase. For high performance liquid chromatography, a mixture of acetonitrile: water (40:60), used as elution and reversed phase column Whatman Partisphere C18 4.6 × 250 mm, used as stationary phase. HPLC was performed with a flow rate of 1 ml/min, pressure: 130 kgF/cm^2^ for 20 min. Thereafter, the solution was filtered through a 0.22 µm filter.

### Determination of partition coefficient

For calculation of partition coefficient (log p), P (ratio of specific activities of organic and aqueous phases) was determined. 37 MBq of the radiolabeled indium complex was added to a mixture of 1 ml of 1-octanol and 1 ml of isotonic acetate buffered saline (pH=7) and then the solution was stirred at 37 °C and kept for 5 min. After that the solution was centrifuged at 1200 g for 5 min. Finally 500 µl of the octanol phase and aqueous phases were sampled and the specific activity in both phases was calculated (20).

### Stability tests

For evaluation of stability test, ITLC method was done. ITLC was carried out for 2 samples and then compared to each other which one is 37 MBq of ^111^In-porphyrin complexes that was kept at room temperature for 2 days and another is a mixture of 500 µl freshly collected human serum and 36.1 MBq (976 µCi) of ^111^In-porphyrin complexes that was incubated at 37 °C for 48 h.

### Biodistribution of labeled compound in wild-type rats

Biodistribution of ^111^In-porphyrin complexes among tissues of wild-type rats were determined. Animals were sacrificed by CO_2_ asphyxiation after injection (2, 4 and 24 h). Dose calibrator with fixed geometry counted the total amount of radioactivity which injected into each rats. The final activity of the radiopharmaceutical for injection was 50 µCi. After that the tissues (blood, heart, lung, brain, intestine, faces, skin, stomach, kidneys, spleen, bone, liver and muscle) were weighed and washed with normal saline. For calculation of percentage of injected dose per gram of tissues, HPGe detector armed with a sample holder device used to determine specific activity of percentage of injected dose per gram of tissues, HPGe detector armed with a sample holder device used to determine specific activity of tissues.

### Imaging of ^111^In-porphyrin complexes in wild-type rats

Images were taken 2, 4 and 24 hours after administration of the radiopharmaceutical by a dual-head SPECT system. The mouse-to-high energy septa distance was 12 cm. Images were taken from both normal and tumor bearing mice. The useful field of view (UFOV) was 540 mm × 400 mm.

## Results and Discussion

### Radionuclide production

Indium-111, in form of InCl_3_, was prepared by 22 MeV proton bombardment of the enriched Cd-112 target at Cyclone-30 on a regular basis. Radionuclidic control showed the presence of 172 and 245 keV gamma energies, all originating from ^111^In and showed a radionuclidic purity higher than 99% (E.O.S.). The concentrations of cadmium (from target material) and copper (from target support) were determined using polarography and shown to be below the internationally accepted levels, i.e. 0.1 ppm for Cd and Cu (21, 22).

The radioisotope was dissolved in acidic media as a starting sample and was further diluted and evaporated for obtaining the desired pH and volume followed by sterile filtering. The radiochemical purity of the In-111 solution was checked in two solvent systems, in 1 mM DTPA, free In^3+^ cation is converted to more lipophilic In-DTPA form and migrates to higher *R*_f_=0.8 while any small radioactive fraction remaining at the origin could be related to other Indium-111 ionic species, not forming In-DTPA complex, such as InCl_4_^-^, etc. and/or colloids (not observed).

**Figure 2:**
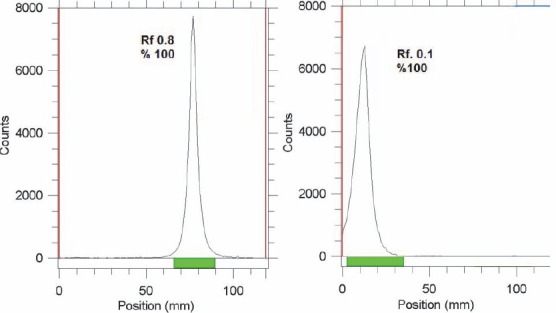
ITLC chromatograms of ^111^InCl_3_ solution in DTPA solution (pH=5) (left) and 10 % ammonium acetate : methanol (1:1) solution (right) using Whatman no. 2 paper

On the other hand, 10 % ammonium acetate:methanol mixture was also used to determine the radiochemical purity. The fast eluting species was possibly the ionic In-111 cations other than In^3+^ (not observed) and the remaining fraction at *R*_f_=0 was a possible mixture of In^3+^ and/or colloids (Figure 2).

**Figure 3:**
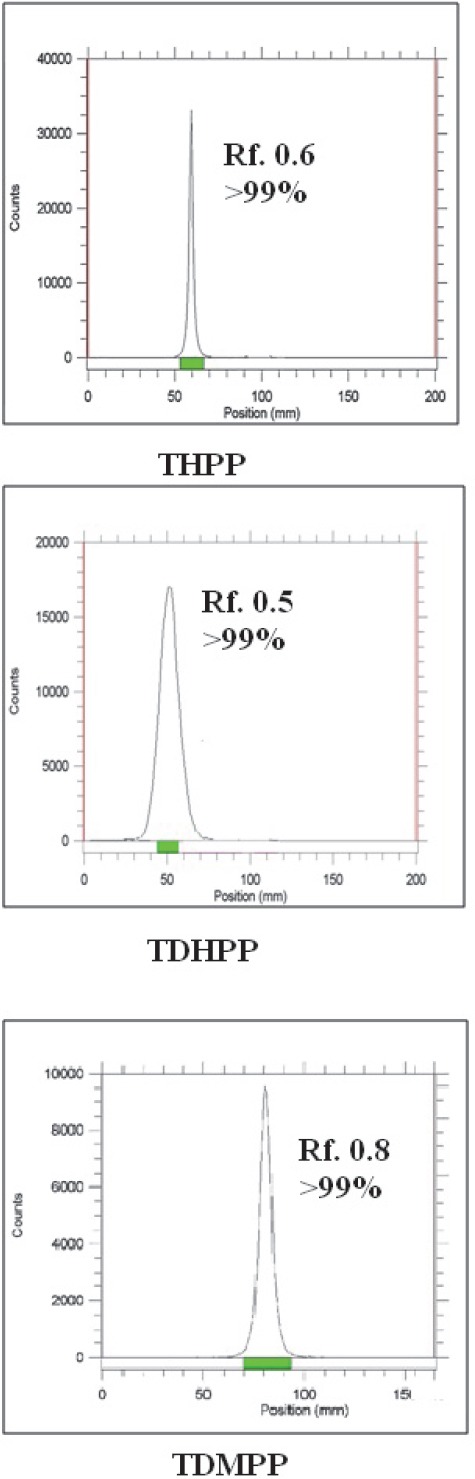
RTLC chromatograms of ^111^In-porphyrins using 10 % ammonium acetate:methanol (1:1) solution (right) on Whatman no. 2

The radiolabeling process was checked by ITLC using 10 % ammonium acetate:methanol (1:1) solution as shown in Figure 3. In all cases radiolabeled porphyrins migrate to higher *R*_f_ due to higher lipophilicity compared to free indium cation. Among the radiolabeled porphyrins In-TDMPP demonstrated higher *R*_f_ due to more lipophilicity compared to two other phenolic derivatives. Dihydroxy complex (^111^In-TDHPP) showed more hydrophilicity compared to the mono hydroxyl compound. ITLC studies approved the production of a single radiolabeled compound in each case.

**Figure 4:**
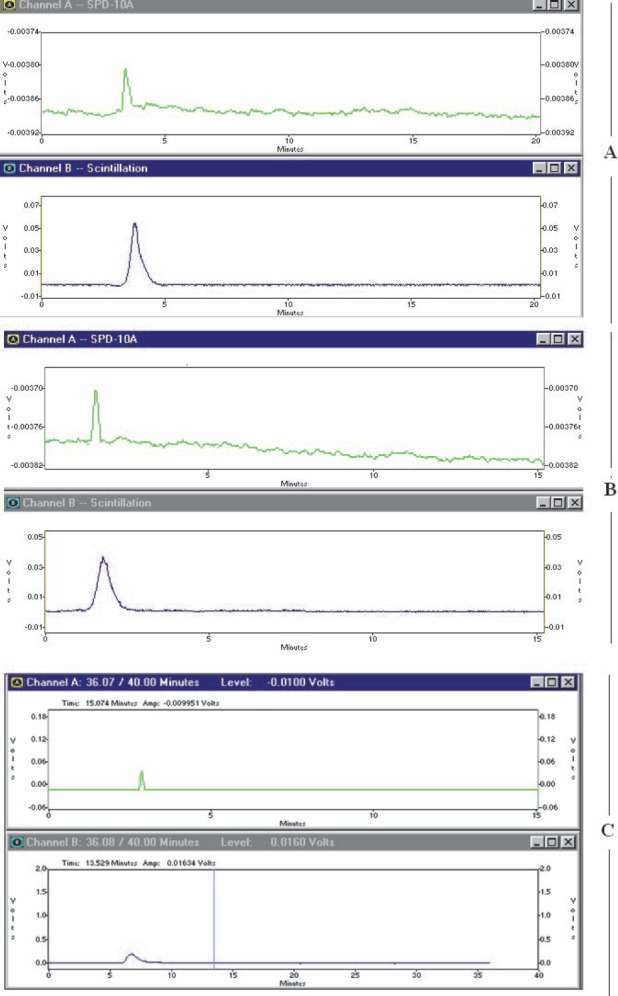
HPLC chromatograms of ^111^In-THPP (section A), ^111^In-TDHPP (section B), ^111^In-TDMPP (section C) on a reversed phase column using acetonitrile:water 40:60, (in all sections; upper: UV chromatogram, scintillation chromatogram; below)

HPLC studies also demonstrated the existence of only one radiolabeled species using both UV and scintillation detectors. In all cases a more fast-eluting compound observed with scintillation detector was almost time- coincident with a related peak of UV detector, demonstrating a more lipophilic compound compared to ^111^In cation (Figure 4).

### Stability studies

Studies on ^111^In-porphyrins showed that stability of the labeled compounds was high enough to carry out further studies. ITLC showed no loss of In-111 from labeled compound after incubation of ^111^In-porphyrins in freshly prepared human serum for 2 days at 37 °C. At physiologic conditions the radiochemical purity of labeled compound remained more than 99% for 2 days.

### Biodistribution studies

**Figure 5:**
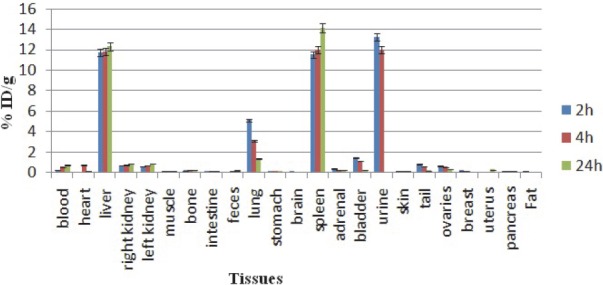
Biodistribution of ^111^InCl_3_ (1.85 MBq, 50 μCi) in wild-type rats 2, 4 and 24 h after injection via tail vein (ID/g%: percentage of injected dose per gram of tissue) (n=5)

**Free indium cation:** For better comparison biodistribution study was performed for free In^3+^. The %ID/g data are summarized in Figure 5.

Indium cation similarity with ferric ion is important in the development of indium radiopharmaceuticals, since iron is an essential element in the human body and a number of iron binding proteins, such as transferrin (in blood), which used in transporting and storing iron *in vivo* (23).

As shown in Figure 5, indium cation almost mimics the ferric cation behavior and is rapidly removed from the circulation and is accumulated in the liver, also a major fraction is excreted rapidly through the kidney leading to high accumulation in urine in 2-24 h post injection as a water soluble cation.

### Radiolabeled porphyrins

**Figure 6:**
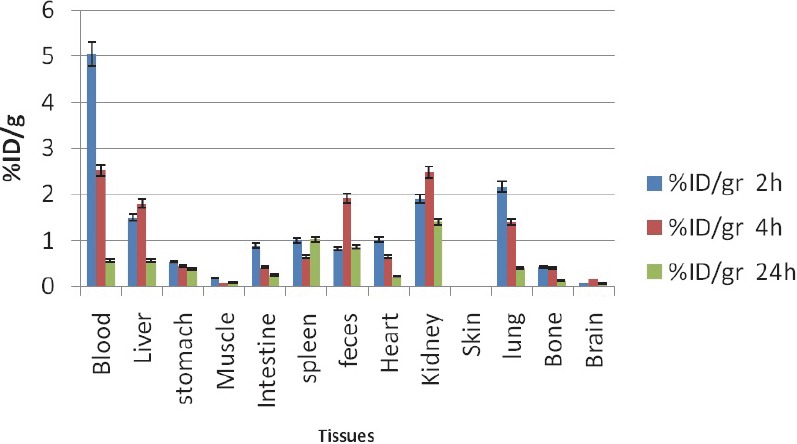
Biodistribution of ^111^In-THPP (50 µCi) in wild type rats 2,4 and 24 h after iv injection via tail vein (ID/g%: percentage of injected dose per gram of tissue calculated based on the area under curve of 245 keV peak in gamma spectrum) (n=3)

**^111^In-THPP:** Biodistribution data of ^111^In-THPP (Figure 6), 2 h after injection the highest activity was seen in and blood pool, while this amount is reduced due to other tissue's uptake. Due to the presence of polar groups in the structure of the compound, a major portion of the radioactive compound was concentrated in kidneys, 2-24 h after injection. Although liver is another accumulation site due to lipoprotein transport into this organ leading to significant feces activity content.

**Figure 7:**
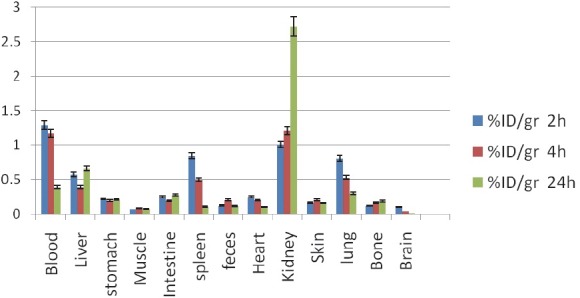
Biodistribution of ^111^In-TDMPP (50 µCi) in wild type rats 2,4 and 24 h after iv injection via tail vein (ID/g%: percentage of injected dose per gram of tissue calculated based on the area under curve of 245 keV peak in gamma spectrum) (n=3)

**^111^In-TDMPP:** despite the presence of two CH_3_O- groups in TMPP complex and low water solubility leading to higher log P, kidneys are the major accumulation sites of excretion while liver stayed a minor excretion route. Lung and spleen also show significant activity (Figure 7).

**Figure 8:**
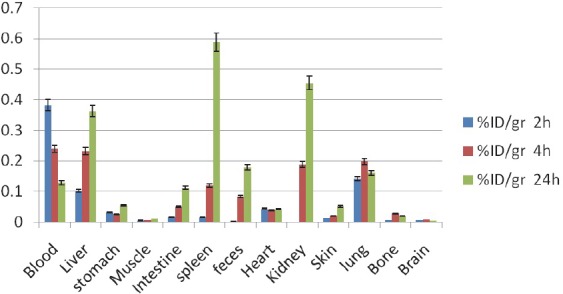
Biodistribution of ^111^In-TDHPP (50 µCi) in wild type rats 2,4 and 24 h after iv injection via tail vein (ID/g%: percentage of injected dose per gram of tissue calculated based on the area under curve of 245 keV peak in gamma spectrum) (n=3)

**^111^In-TDHPP:** Similar to ^111^In-THPP, *di*-hydroxy compound is also accumulated majorly in the liver and kidneys which are typical accumulation sites for porphyrins. Due to oxidative/reductive enzymes present in lungs a significant uptake is also observed (Figure 8).

Due to the importance of low liver accumulation compared to the kidneys excretion, a kidney: liver uptake ratio can be suggested as a suitable criterion for rapid clearance of the radiolabeled complexes as tumor imaging agents, while imposing less irradiation dose as shown in Table 1.

**Table 1:** The kidney: liver uptake ratio of the radiolabeled complexes in 2, 4 and 24 h post injection (n=3)

Time/complex	^111^In-TDHPP	^111^In-THPP	^111^In-TDMPP
**2 h**	1.46	1.27	1.74
**4 h**	2.08	1.37	3.08
**24 h**	1.65	2.47	4.13

As shown in Table 1, TDMPP complex demonstrates the best kidney:liver uptake ratio among the three complexes at all time intervals despite the high lipophilic behavior observed in partition coefficient and chromatographic studies.

It can be proposed that a metabolic pathway leading to the formation of more water soluble metabolites exists for *di*-methoxy complex. Thus ^111^In-TDMPP complex is probably a suitable candidate for considering as a possible tumor imaging agent.

### Imaging of wild-type rats

**Figure 9:**
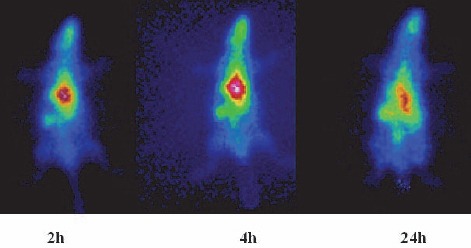
SPECT images of ^111^In-THPP (70 μCi) in wild-type rats 2, 4 and 24 h post injection

The In-111 labeled porphyrin imaging in the wild-type rats showed a distinct accumulation of the radiotracer in the chest region all the time after injection. Most of the activity is washed out from the body after 24 h and the picture contrast weakened. Figure 9, demonstrates the liver and spleen uptake of ^111^In-THPP complex among the rat tissues at all time intervals, however a minor kidney uptake is also observed. These findings are in full agreement with the dissection studies shown in Figure 6.

**Figure 10:**
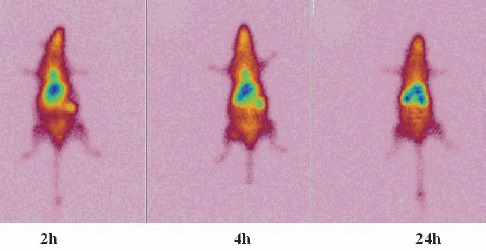
SPECT images of ^111^In-TDMPP (70 μCi) in wild-type rats 2, 4 and 24 h post injection

In case of ^111^In-TDMPP, kidneys are the significant excreting organs although lung, liver and spleen uptakes are observed in a single signal (Figure 10). These findings are in full agreement with the dissection studies shown in Figure 7.

**Figure 11:**
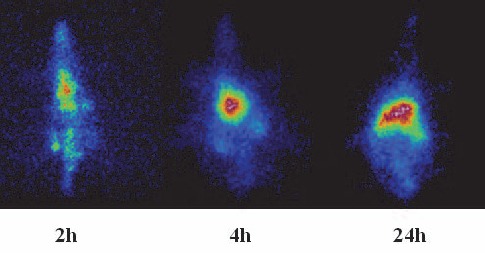
SPECT images of ^111^In-TDHPP (70 μCi) in wild-type rats 2, 4 and 24 h post injection

Figure 11, also demonstrates the liver and spleen uptake of ^111^In-TDHPP complex among the rat tissues at all time intervals. A significant kidney uptake is also observed. These findings are in agreement with the dissection studies shown in Figure 8.

All porphyrins are transferred in the body as bonded to the lipoproteins, thus any possible targets of lipoproteins are the accumulation site, and among many other porphyrins this is the major drawback.

## Conclusion

^111^In-TDHPP, ^111^In-THPP and ^111^In-TDMPP were prepared using ^111^InCl_3_ (produced from proton bombardment of ^nat^Cd target) in 60 min at 80 ºC. The complexes were prepared with more than 99% radiochemical purity (HPLC and RTLC) and high stability to 48h. Partition coefficients (calculated as log P) for ^111^In-TDHPP, ^111^In-THPP and ^111^In-TDMPP were 0.88, 0.8 and 1.63 respectively. Biodistribution studies using SPECT imaging and tissue dissection demonstrated significant urinary excretion with fast clearance for ^111^In-TDMPP, while the two other complexes demonstrated liver uptake and longer retention in animal tissues. Thus ^111^In-TDMPP complex is probably a suitable candidate for considering as a possible tumor imaging agent.
